# Case Report: Successful treatment of new onset plaque psoriasis with secukinumab in a peritoneal dialysis patient

**DOI:** 10.3389/fimmu.2026.1882041

**Published:** 2026-07-08

**Authors:** Junyan Fang, Ruolin Li, Yingxin Xie, Yingli Liu

**Affiliations:** Department of Nephrology, Shanghai Ninth People’s Hospital, School of Medicine, Shanghai Jiao Tong University, Shanghai, China

**Keywords:** autoimmune disease, case report, peritoneal dialysis, psoriasis, secukinumab

## Abstract

Psoriasis is an immune-mediated inflammatory skin disorder associated with systemic complications, including an elevated risk of chronic kidney disease (CKD) and progression to end-stage renal disease (ESRD). Managing psoriasis in patients undergoing peritoneal dialysis (PD) remains challenging, primarily due to contraindications to conventional systemic therapies and the limited evidence regarding biologic agents in this population. We report a case of severe psoriasis successfully treated with secukinumab in a 61-year-old man on PD. The patient was diagnosed with plaque psoriasis six years after dialysis initiation. Although initial symptoms improved with intensified dialysis and topical corticosteroids, the disease subsequently was exacerbated, presenting with extensive lesions and severe generalized pruritus that significantly impaired sleep quality. Secukinumab was initiated at 300 mg subcutaneously weekly for four weeks, followed by a maintenance dose of 300 mg every four weeks. At 12 months, the patient achieved complete clearance of psoriatic lesions with no adverse events or PD-related complications. This case highlights the paradoxical role of dialysis in psoriasis, which may either induce new-onset disease or ameliorate pre-existing lesions. Furthermore, it supports the efficacy and safety of secukinumab for severe psoriasis in PD patients. While IL-17A is implicated in dialysis-induced peritoneal damage, whether IL-17A inhibitors confer protective benefits on peritoneal membrane integrity in this population remains unknown and warrants dedicated investigation. Further studies are urgently needed to establish evidence-based guidelines for secukinumab use in PD patients.

## 1 Introduction

Psoriasis is a chronic, immune-mediated inflammatory skin disease affecting 2–3% of the global population, with profound impacts on physical and psychological well-being. Its pathogenesis involves uncontrolled keratinocyte proliferation and immune system dysregulation, primarily mediated by the IL-23/Th17 axis, and manifests as distinct clinical phenotypes, including plaque psoriasis (the most common form), as well as guttate, erythrodermic, and pustular subtypes (1, 2).

Beyond cutaneous manifestations, psoriasis is now recognized as a systemic inflammatory disease, generating a wide array of pro-inflammatory mediators both locally in the lesions and systemically in the circulation (3, 4). This systemic inflammation contributes to various comorbidities, such as diabetes mellitus, cardiometabolic disorders, gastrointestinal diseases, obesity, and chronic kidney disease (CKD) (3, 4). Moreover, the relationship between psoriasis and CKD may be bidirectional (5–7). Meta-analyses have demonstrated that patients with psoriasis, especially those with severe disease, face an increased risk of developing CKD (pooled HR 1.91, 95% CI 1.78–2.05) and end-stage renal disease (ESRD) (pooled HR 2.72, 95% CI 1.70–4.34) (5). Severe psoriasis not only accelerates the progression of CKD to ESRD but also carries a fourfold higher risk of kidney-related mortality (6–8). Conventional systemic therapies pose additional challenges in psoriasis patients with renal impairment. Methotrexate and cyclosporine frequently require dose adjustments due to their nephrotoxicity; notably, even low-dose methotrexate can precipitate severe adverse events, including death, in patients with CKD (9, 10). These factors collectively create unique therapeutic dilemmas in the management of psoriasis complicated by renal impairment.

Biologic agents, particularly the IL-17A inhibitor secukinumab, have emerged as a promising alternative for challenging psoriasis cases. Although evidence supports their efficacy and safety in psoriasis patients receiving hemodialysis (HD) (11–13), data for the peritoneal dialysis (PD) population are notably lacking. Interestingly, dialysis itself can improve psoriatic skin lesions, and PD may confer advantages over HD in this regard (14, 15). Here, we describe a case of *de novo* plaque psoriasis developing six years after PD initiation, which was successfully treated with secukinumab. Our experience emphasizes both the feasibility of biologic therapy in PD patients and the critical need for systematic studies to guide clinical decision-making in this population.

## 2 Case presentation

A 61-year-old man with ESRD secondary to hypertension and type 2 diabetes mellitus had been treated with continuous ambulatory peritoneal dialysis (CAPD) since January 2017. Following the loss of residual renal function and the development of refractory edema, his dialysis regimen was intensified with higher-glucose-concentration solutions.

**Figure 1:**
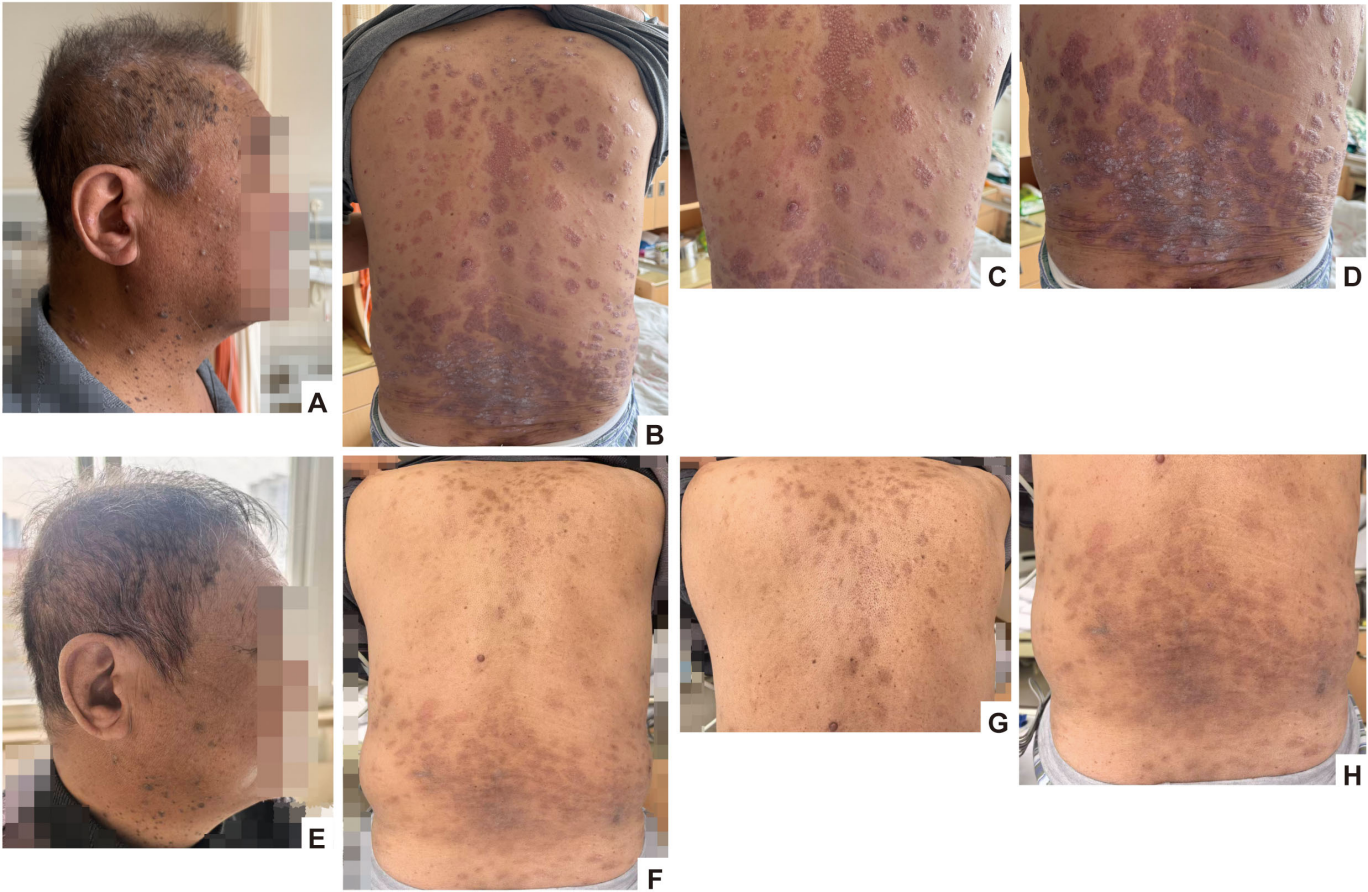
Clinical appearance of the patient. **(A–D)** Before secukinumab treatment. **(E–H)** After 10 months of secukinumab treatment.

In May 2023, he presented with small, well-demarcated, erythematous, scaly plaques on his back. Dermatological evaluation confirmed plaque psoriasis, with a Psoriasis Area and Severity Index (PASI) score of 1.8. The condition was initially managed with topical corticosteroids. At that time, dialysis adequacy was suboptimal (urea clearance index [Kt/V] 1.34; creatinine clearance rate [CCR] 43.67 L/week/1.73 m²). To optimize solute clearance, his therapy was intensified by transitioning to automated PD (APD), which resulted in transient improvement of the psoriatic lesions over the following two months. However, at the six-month follow-up, despite achieving adequate dialysis targets (Kt/V 1.78; CCR 53.05 L/week/1.73 m²), the patient experienced a recurrence and progression of psoriasis. The lesions evolved into extensive, confluent, erythematous plaques with thick, silvery-white scales, involving the entire back, trunk, and scalp (**Figures 1A–D**), with a PASI score of 14. He reported severe generalized pruritus that significantly impaired his sleep quality. He denied any joint pain or swelling. There was no family history of psoriasis, and no new medications had been introduced.

Laboratory findings were as follows: leukocyte count 5,900/µL (neutrophils 76.8%, lymphocytes 11.3%, monocytes 8.1%, eosinophils 3.1%), hemoglobin 12.0 g/dL, and C-reactive protein 13.6 mg/L. Hemoglobin A1c was 10%. Liver function tests, hepatitis B and C serologies, syphilis screening (TRUST), HIV screening, and T-SPOT.TB results were all within normal limits. Computed tomography showed no evidence of infection or malignancy.

**Figure 2:**
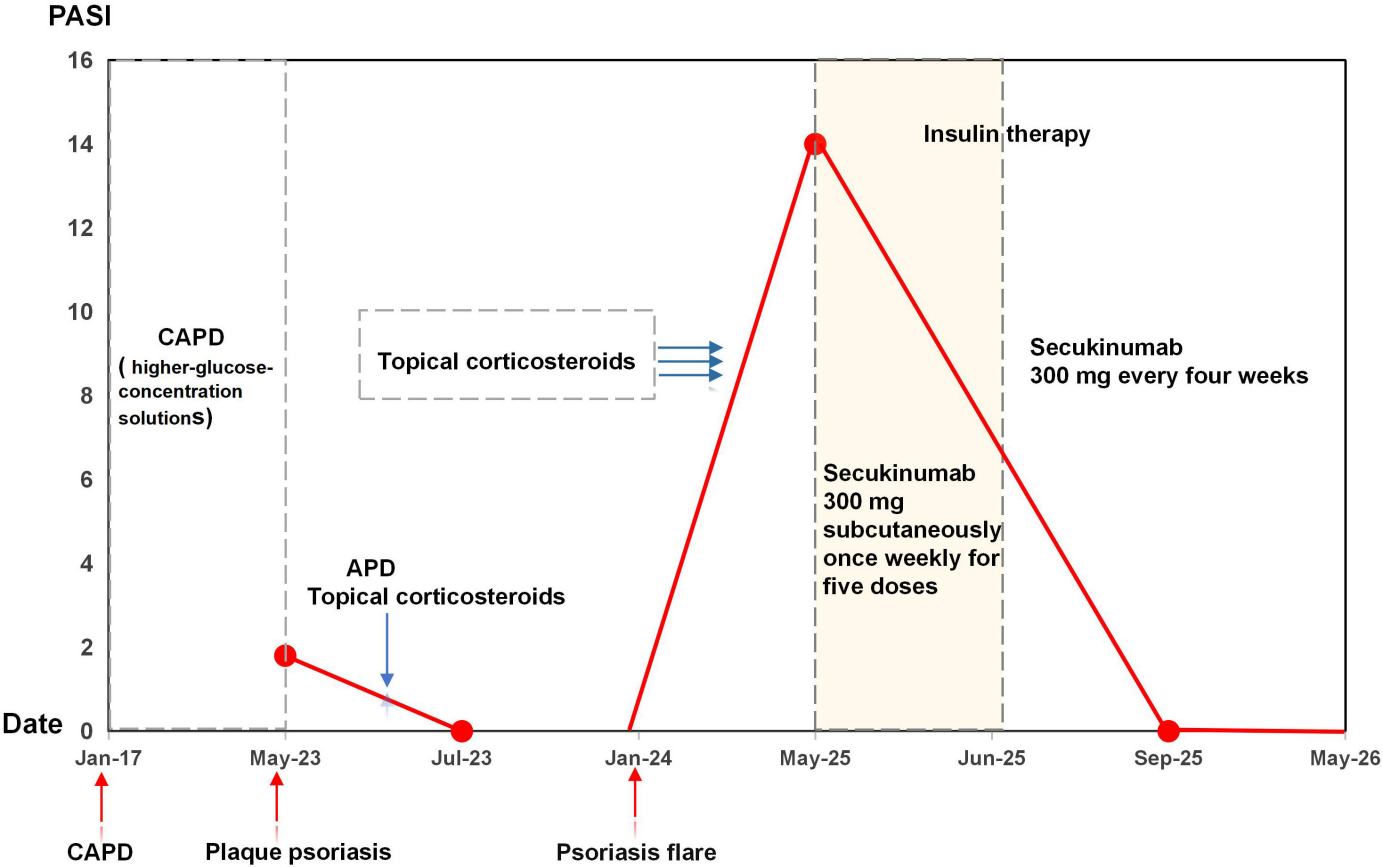
Flowchart summarizing the patient’s clinical course and treatment timeline.

Following multidisciplinary consultation and informed consent for off-label use, the standard secukinumab regimen was initiated (300 mg subcutaneously once weekly for five doses [Weeks 0, 1, 2, 3, and 4], followed by 300 mg every four weeks). Concomitantly, insulin therapy was intensified to optimize glycemic control. The patient’s HbA1c improved from 10.0% at baseline to 8.2% at month 6. By the four-week follow-up, the patient reported significant subjective improvement and a marked reduction in psoriatic skin lesions, and the treatment was well tolerated. By month 4, the patient achieved complete clearance of psoriasis (PASI 0), with no recurrence or adverse events observed during the subsequent 12-month follow-up (**Figures 1E–H**). The patient continued PD with his original prescription. Following secukinumab initiation, the peritoneal solute transport rate (PSTR) increased (e.g., 4-hour D/P creatinine rose from 0.64 to 0.73); however, dialysis adequacy (Kt/V 1.95; CCR 53.43 L/week/1.73 m²) and ultrafiltration capacity remained well preserved. The post-treatment serum IL-17A level was 6.15 pg/mL (reference range: ≤ 8.60 pg/mL). A flowchart summarizing the patient’s clinical course and treatment timeline is shown in **Figure 2**.

## 3 Discussion

Dialysis is associated with various dermatological conditions; however, psoriasis remains relatively uncommon among dialysis patients, with a prevalence of approximately 2.7% (16). While several case reports have documented new-onset psoriasis in patients with ESRD following the initiation of either HD or PD (16–18), a retrospective cohort study demonstrated that ESRD patients on chronic HD face an elevated risk of developing psoriasis compared with a matched control population (19). Conversely, numerous studies have reported significant improvement in severe or refractory psoriasis following dialysis therapy, even in patients without renal impairment (14, 15). Moreover, PD appears to be associated with higher rates of lesion clearance compared with HD (14, 15). Consequently, dialysis exhibits a paradoxical role in the context of psoriasis. Illustrating this paradox, our patient developed psoriasis six years after initiating PD therapy, a finding consistent with previous reports of dialysis-associated onset (16–18). The skin lesions resolved transiently following intensive dialysis and topical corticosteroid therapy.

Another plausible explanation for the psoriasis flare in this case is poorly controlled hyperglycemia. Elevated blood glucose levels are significantly correlated with disease severity in psoriasis patients (20). Notably, an HbA1c level ≥ 7% has been identified as an independent prognostic factor for disease exacerbation in patients with severe psoriasis and concurrent diabetes or prediabetes (21). Mechanistically, hyperglycemia stimulates the secretion of pro-inflammatory cytokines, such as TNF-α and IL-6, which are well-established drivers of psoriasis pathogenesis (20, 22). In our specific case, the intensification of dialysis—particularly the use of high-glucose PD fluid—precipitated severe hyperglycemia (HbA1c 10%) and likely promoted the accumulation of advanced glycation end products (AGEs). These AGEs can induce keratinocyte proliferation and enhance innate immune activation, thereby exacerbating psoriatic inflammation (23, 24).

The clinical importance of glycemic control is further underscored by existing literature. A prospective analysis by Enos et al. demonstrated that diabetes is independently associated with a poorer response to biologics; after adjusting for confounders, the likelihood of achieving PASI 75 was reduced by approximately 31% (OR, 0.69; 95% CI, 0.56-0.85) (25). Conversely, Sun et al. reported that psoriasis patients treated with hypoglycemic agents exhibited significant reductions in PASI scores and an increased likelihood of achieving PASI 75 (26). Therefore, rigorous management of hyperglycemia in patients with diabetic psoriasis may reduce the frequency of flares, alleviate disease severity, and improve therapeutic responses.

Biologic agents, including anti-TNF-α agents, anti-IL-12/23 therapies, IL-17A inhibitors, and IL-17R blockers, have revolutionized the management of psoriasis. However, data regarding their efficacy and safety in psoriasis patients with ESRD undergoing dialysis remain limited (27). For instance, Sandys et al. reported a case of acute kidney injury secondary to anti-TNF-α therapy (28). Furthermore, Chen et al. observed that among patients with severe psoriasis, those receiving anti-TNF-α or anti-IL-12/23 therapies experienced progression of CKD over time, whereas patients treated with anti-IL-17 biologics maintained stable CKD stages (29). As summarized in **Table 1**, the IL-17 inhibitors secukinumab and ixekizumab have demonstrated promising efficacy and safety profiles in dialysis patients with various forms of psoriasis (11–13, 30–33). Given that preserving residual kidney function is critically important for patients undergoing PD, we favor anti-IL-17 biologics for this population.

**Table 1:** Reported cases of IL-17 inhibitors in psoriasis patients on dialysis.

References	Age/sex	Dialysis type	Disease	Biologic	Outcome	Adverse event
Pizzatti L et al. (11)	44/M	hemodialysis	Erythrodermic psoriasis	secukinumab	PASI-100 response at week 8 (PASI 31.5 to PASI 0)	None
Xiao Y et al. (12)	48/M	hemodialysis	plaque psoriasis	Secukinumab	PASI-100 response at week 8 (PASI 19.2 to PASI 0)	None
Mukai M et al. (13)	59/M	Peritoneal dialysis	Erythrodermic psoriasis and arthritis	Secukinumab	PASI-90 response after 5 months (PASI 33 to unknown)	None
	60/M	hemodialysis	Psoriasis vulgaris	Secukinumab	PASI-90 response after 4 months (PASI 31 to unknown)	None
	49/M	hemodialysis	psoriatic arthritis	Secukinumab	PASI-90 response (PASI unknown)	None
Ikuma D et al. (30)	60/F	hemodialysis	plaque psoriasis	Secukinumab	PASI-100 response at week 6 (PASI 49.8 to PASI 0)	None
Shibata T et al. (31)	61/F	hemodialysis	Erythrodermic psoriasis	Secukinumab	PASI-100 response after 2 months (PASI 22 to PASI 0)	None
Zhang S et al. (32)	67/M	hemodialysis	Erythrodermic psoriasis	Secukinumab	PASI-95 response after one month (PASI 46.2 to PASI 2.4)	None
Zhu X et al. (33)	39/F	peritoneal dialysis	plaque psoriasis	Ixekizumab	PASI-100 response after 4 months (PASI 16 to PASI 0)	None

Beyond renal protection, targeting IL-17 may offer additional pathophysiological benefits specific to PD. Chronic exposure to conventional PD fluid promotes the recruitment of various IL-17A-producing cells and augments cytokine production (34, 35). Mechanistically, IL-17A activates peritoneal cells to upregulate pro-inflammatory cytokines and profibrotic factors, such as IL-6 and CX3CL1, thereby driving sustained inflammation and peritoneal fibrosis (34, 35). Furthermore, treatment with an IL-17A neutralizing antibody has been shown to successfully ameliorate peritoneal damage induced by PD fluid (34, 36). This pathophysiological overlap between psoriasis and PD-induced peritoneal damage provides a compelling rationale for utilizing IL-17 inhibition in psoriasis patients on PD. In the present case, peritoneal membrane function was evaluated using the peritoneal equilibration test (PET). Following 12 months of secukinumab therapy, PET revealed an increased PSTR. Notably, both dialysis adequacy (as evidenced by an increase in Kt/V from 1.78 to 1.95) and ultrafiltration capacity remained well preserved. Based on these data, we cannot definitively conclude that targeting IL-17A in psoriasis treatment confers protective effects on the peritoneum, a conclusion that would ideally require comparing pre- and post-treatment peritoneal biopsies.

As a first-in-class, fully human monoclonal antibody (IgG1-κ), secukinumab undergoes degradation into small peptides and amino acids via intracellular catabolic pathways (37), similar to endogenous IgG. Consequently, renal impairment is not expected to significantly alter its systemic exposure. However, the safety and efficacy of secukinumab have not been formally evaluated in patients with severe renal impairment, as this population was excluded from pivotal psoriasis clinical trials based on protocol-specified criteria (e.g., serum creatinine > 176.8 µmol/L) (38). Given documented cases of successful and well-tolerated use of secukinumab in HD patients (11–13, 30–32), a standard dosing regimen was adopted for our patient on PD.

Several clinical considerations warrant emphasis. First, no published data are currently available on the pharmacokinetics of secukinumab in patients undergoing chronic dialysis. Evidence suggests that peritoneal losses of IgG in patients undergoing CAPD are influenced by the PSTR and dialysate dwell time (39, 40). Consequently, PD may alter serum concentrations of secukinumab, highlighting the need to monitor its pharmacokinetic profile. Larger prospective studies are required to further characterize its pharmacokinetics in this population. Second, vigilant monitoring for adverse events is imperative. Although secukinumab is generally well tolerated, clinicians should remain alert to potential risks, such as Candida infection, uveitis, and malignancy (41), particularly in immunocompromised individuals, including those on dialysis. Candida species are the most common pathogens implicated in fungal peritonitis (FP), a rare but serious infection associated with a mortality rate of 20–30% (42). Accordingly, the 2022 ISPD guidelines strongly recommend immediate catheter removal in cases of FP (42).

Several limitations of this study should be acknowledged. First, the patient’s glycemic control did not reach the target level (HbA1c < 7%). Consequently, more intensive dietary education and stricter adherence to glucose-lowering therapy would have been required to avoid compromising the therapeutic response to psoriasis, highlighting the need for comprehensive metabolic management in future cases. Second, while the clinical improvement in psoriasis was striking and the post-treatment serum IL-17 level was within the normal range, we did not measure baseline IL-17 levels. Therefore, we cannot definitively demonstrate that IL-17A activity was elevated before treatment or that secukinumab effectively suppressed it. However, the robust clinical response remains consistent with existing literature. Third, previous studies have demonstrated that blocking IL-17A alleviates peritoneal damage induced by PD fluids. In our case, however, the PSTR increased from a D/P creatinine of 0.64 to 0.73 after 12 months of secukinumab therapy, which does not directly support this hypothesized protective effect. Without pre- and post-treatment peritoneal biopsies, we cannot determine whether this change was attributable to secukinumab, the natural progression of peritoneal membrane function over time, or other clinical variables (e.g., peritoneal glucose exposure). Consequently, the potential peritoneal protective effects of secukinumab in psoriasis patients undergoing PD remain merely hypothetical. Finally, although our case demonstrates the efficacy and safety of secukinumab in psoriasis patients undergoing PD, this is a single case report with a relatively short follow-up period, and the findings may not be generalizable to the broader PD population. Despite these limitations, the observed complete and sustained clearance of psoriasis with no adverse events or dialysis-related complications provides valuable real-world evidence for the use of secukinumab in this understudied patient population.

## 4 Conclusion

This case demonstrates the rapid efficacy and favorable safety profile of secukinumab in a patient with psoriasis and ESRD who required PD. Targeting IL-17A offers a promising therapeutic approach for psoriasis in PD patients; however, potential benefits to peritoneal function remain unproven and warrant further study. Nevertheless, the paucity of pharmacokinetic data, the elevated risk of infection, and the limitations noted above necessitate a prudent clinical approach. Therefore, prospective multicenter trials are essential to establish evidence-based guidelines for secukinumab in this vulnerable population.

## Data Availability

The raw data supporting the conclusions of this article will be made available by the authors, without undue reservation.

## References

[1] Armstrong AW Blauvelt A Callis Duffin K Huang YH Savage LJ Guo L . Psoriasis. Nat Rev Dis Primers. (2025) 11:45. doi: 10.1038/s41572-025-00630-5 40571687

[2] Griffiths CEM Armstrong AW Gudjonsson JE Barker JNWN . Psoriasis. Lancet. (2021) 397:1301–15. doi: 10.1016/S0140-6736(20)32549-6 33812489

[3] Scala E Mercurio L Albanesi C Madonna S . The intersection of the pathogenic processes underlying psoriasis and the comorbid condition of obesity. Life (Basel). (2024) 14:733. doi: 10.3390/life14060733 38929716 PMC11204971

[4] Mrowietz U Lauffer F Sondermann W Gerdes S Sewerin P . Psoriasis as a systemic disease. Dtsch Arztebl Int. (2024) 121:467–72. doi: 10.3238/arztebl.m2024.0064 38657176 PMC11635804

[5] Jing X Zhuyuan W Aijun C Jianxia X Kun H Ping W . Association of psoriasis with chronic kidney disease and end-stage renal disease: a systematic review and meta-analysis. Front Med (Lausanne). (2023) 10:1175477. doi: 10.3389/fmed.2023.1175477 37250627 PMC10213311

[6] Lu J Li H Wang S . Interaction effect of psoriasis and chronic kidney disease on the risk of all-cause mortality: a prospective cohort study of NHANES data. Nephrol Dial Transplant. (2023) 38:2474–84. doi: 10.1093/ndt/gfad089 37173279

[7] Chi CC Wang J Chen YF Wang SH Chen FL Tung TH . Risk of incident chronic kidney disease and end-stage renal disease in patients with psoriasis: a nationwide population-based cohort study. J Dermatol Sci. (2015) 78:232–8. doi: 10.1016/j.jdermsci.2015.03.012 25862150

[8] Abuabara K Azfar RS Shin DB Neimann AL Troxel AB Gelfand JM . Cause-specific mortality in patients with severe psoriasis: a population-based cohort study in the U.K. Br J Dermatol. (2010) 163:586–92. doi: 10.1111/j.1365-2133.2010.09941.x 20633008 PMC2966545

[9] Ouellette S Shah R Razi S Ashforth G Wassef C . Fatal low-dose methotrexate toxicity: a case report and literature review. Dermatol Ther. (2022) 35:e15945. doi: 10.1111/dth.15945 36259229

[10] Muanda FT Blake PG Weir MA Ahmadi F McArthur E Sontrop JM . Low-dose methotrexate and serious adverse events among older adults with chronic kidney disease. JAMA Netw Open. (2023) 6:e2345132. doi: 10.1001/jamanetworkopen.2023.45132 38010652 PMC10682837

[11] Pizzatti L Mugheddu C Sanna S Atzori L Rongioletti F . Erythrodermic psoriasis in a dialyzed patient successfully treated with secukinumab. Dermatol Ther. (2020) 33:e13348. doi: 10.1111/dth.13348 32239791

[12] Xiao Y Sun J . Successful treatment with secukinumab in a psoriasis patient on hemodialysis. Psoriasis (Auckl). (2025) 15:321–5. doi: 10.2147/PTT.S536639 40718360 PMC12296631

[13] Mukai M Kurihara Y Ito Y Shintani Y Takahashi H Kubo A . Successful treatment with secukinumab of three psoriatic patients undergoing dialysis. J Dermatol. (2020) 47:e26-e28. doi: 10.1111/1346-8138.15132 31657047

[14] Pandey P Kumar S . Effectiveness of dialysis in psoriasis: a short review. Cureus. (2022) 14:e30061. doi: 10.7759/cureus.30061 36381899 PMC9637456

[15] Sobh MA Abdel Rasik MM Moustafa FE el-Sharabasy MM Rezk RA el-Shamy SI . Dialysis therapy of severe psoriasis: a random study of forty cases. Nephrol Dial Transplant. (1987) 2:351–8. doi: 10.1093/oxfordjournals.ndt.a091573 3122113

[16] Rimsevicius L Sukackiene D Tamulyte G Kirkilaite G Miglinas M . Psoriasis in a patient on peritoneal dialysis: a two-sided mirror. Iran J Kidney Dis. (2017) 11:70–3. 28174356

[17] Geerse DA Suijkerbuijk J van Poppelen KM Litjens EJ Cornelis T . New-onset psoriasis during peritoneal dialysis. Perit Dial Int. (2014) 34:802–3. doi: 10.3747/pdi.2013.00249 25520486 PMC4269508

[18] AlKindi F Saedi MA Boobes Y . New onset plaque psoriasis in a hemodialysis patient: a case report and review of literature. Indian J Nephrol. (2024) 34:195–7. doi: 10.4103/ijn.ijn_57_22 38681003 PMC11044658

[19] Wang CC Tang CH Huang KC Huang SY Sue YM . Increased risk of incident psoriasis in end-stage renal disease patients on chronic hemodialysis: a nationwide population-based cohort study. J Dermatol. (2018) 45:1063–70. doi: 10.1111/1346-8138.14531 29993144

[20] Ikumi K Odanaka M Shime H Imai M Osaga S Taguchi O . Hyperglycemia is associated with psoriatic inflammation in both humans and mice. J Invest Dermatol. (2019) 139:1329–1338.e7. doi: 10.1016/j.jid.2019.01.029 30776434

[21] Yongpisarn T Thadanipon K Suchonwanit P Rattanakaemakorn P . Hyperglycemia is a potential prognostic factor for exacerbation in severe psoriasis with diabetes or prediabetes. Clin Cosmet Investig Dermatol. (2025) 18:345–53. doi: 10.2147/CCID.S502333 39931717 PMC11809405

[22] Chiu HY Hung CJ Muo CH Fan KC Sung FC . The bidirectional association between type 2 diabetes and psoriasis: two retrospective cohort studies. Indian J Dermatol Venereol Leprol. (2020) 86:366–74. doi: 10.4103/ijdvl.IJDVL_428_18 32031110

[23] Papagrigoraki A Maurelli M Del Giglio M Gisondi P Girolomoni G . Advanced glycation end products in the pathogenesis of psoriasis. Int J Mol Sci. (2017) 18:2471. doi: 10.3390/ijms18112471 29156622 PMC5713437

[24] Kang P Chen J Wang S Zhang S Li S Guo S . Advanced glycation end products-induced activation of keratinocytes: a mechanism underlying cutaneous immune response in psoriasis. J Innate Immun. (2023) 15:876–92. doi: 10.1159/000534639 37989127 PMC10715758

[25] Enos CW Ramos VL McLean RR Lin TC Foster N Dube B . Comorbid obesity and history of diabetes are independently associated with poorer treatment response to biologics at 6 months: a prospective analysis in Corrona Psoriasis Registry. J Am Acad Dermatol. (2022) 86:68–76. doi: 10.1016/j.jaad.2021.06.883 34256035

[26] Sun X Cai X Liu L Li H Zhou Y Luo Y . Effect of different types of hypoglycemic medications on psoriasis: an analysis of current evidence. Dermatology. (2023) 239:299–313. doi: 10.1159/000528026 36623489

[27] Geng RSQ Sood S Waked J Merchant N Abduelmula A Yeung J . Biologic therapy in psoriasis patients with renal disease: a systematic review. J Cutan Med Surg. (2025) 29:194–5. doi: 10.1177/12034754241293085 39450647

[28] Sandys V Moloney B Lane L Qazi J Doyle B Barry M . Granulomatous interstitial nephritis secondary to adalimumab therapy. Clin Kidney J. (2018) 11:219–21. doi: 10.1093/ckj/sfx104 29644062 PMC5887274

[29] Chen CB Huang YT Hsiao CC Chang SH Chi CC . Real-world effects of biologics on renal function in psoriatic patients: a retrospective study. BioDrugs. (2022) 36:657–66. doi: 10.1007/s40259-022-00547-5 35994233

[30] Ikuma D Oguro M Hoshino J Mizuno H Sekine A Kawada M . Efficacy of secukinumab for plaque psoriasis in a patient on hemodialysis. CEN Case Rep. (2020) 9:55–8. doi: 10.1007/s13730-019-00426-z 31654299 PMC6990267

[31] Shibata T Muto J Takama H Yanagishita T Ito T Watanabe D . Case of psoriatic erythroderma induced by the discontinuation of the chronic use of topical steroid after dialysis initiation and successfully treated with secukinumab. J Dermatol. (2019) 46:e119-e120. doi: 10.1111/1346-8138.14649 30230569

[32] Zhang S Liu P Liu S He M Zheng S Sun X . Successful treatment with secukinumab in an erythrodermic psoriasis patient with end-stage kidney disease on hemodialysis: a case report. Clin Cosmet Investig Dermatol. (2025) 18:2829–33. doi: 10.2147/CCID.S545123 41180113 PMC12577450

[33] Zhu X Pan X Dong Z . Plaque psoriasis with renal dysfunction successfully treated with ixekizumab. Hemodial Int. (2025) 29:126–9. doi: 10.1111/hdi.13185 39397268

[34] Rodrigues-Díez R Aroeira LS Orejudo M Bajo MA Heffernan JJ Rodrigues-Díez RR . IL-17A is a novel player in dialysis-induced peritoneal damage. Kidney Int. (2014) 86:303–15. doi: 10.1038/ki.2014.33 24552849

[35] Helmke A Hüsing AM Gaedcke S Brauns N Balzer MS Reinhardt M . Peritoneal dialysate-range hypertonic glucose promotes T-cell IL-17 production that induces mesothelial inflammation. Eur J Immunol. (2021) 51:354–67. doi: 10.1002/eji.202048733 32926407

[36] Marchant V Tejera-Muñoz A Marquez-Expósito L Rayego-Mateos S Rodrigues-Diez RR Tejedor L . IL-17A as a potential therapeutic target for patients on peritoneal dialysis. Biomolecules. (2020) 10:1361. doi: 10.3390/biom10101361 32987705 PMC7598617

[37] Blair HA . Secukinumab: a review in psoriatic arthritis. Drugs. (2021) 81:483–94. doi: 10.1007/s40265-021-01476-3 33661486 PMC8049904

[38] Langley RG Elewski BE Lebwohl M Reich K Griffiths CE Papp K . Secukinumab in plaque psoriasis--results of two phase 3 trials. N Engl J Med. (2014) 371:326–38. doi: 10.1056/NEJMoa1314258 25007392

[39] Cueto-Manzano AM Gamba G Correa-Rotter R . Quantification and characterization of protein loss in continuous ambulatory peritoneal dialysis. Rev Invest Clin. (2000) 52:611–7. doi: 10.1016/b978-1-4160-6193-9.10065-x 11256103

[40] Cueto-Manzano AM Gamba G Correa-Rotter R . Peritoneal protein loss in patients with high peritoneal permeability: comparison between continuous ambulatory peritoneal dialysis and daytime intermittent peritoneal dialysis. Arch Med Res. (2001) 32:197–201. doi: 10.1016/s0188-4409(01)00271-5 11395184

[41] Langley RG Sofen H Dei-Cas I Reich K Sigurgeirsson B Warren RB . Secukinumab long-term efficacy and safety in psoriasis through to year 5 of treatment: results of a randomized extension of the phase III ERASURE and FIXTURE trials. Br J Dermatol. (2023) 188:198–207. doi: 10.1093/bjd/ljac040 36763857

[42] Li PK Chow KM Cho Y Fan S Figueiredo AE Harris T . ISPD peritonitis guideline recommendations: 2022 update on prevention and treatment. Perit Dial Int. (2022) 42:110–53. doi: 10.1177/08968608221080586 35264029

